# Hybrid Biogeography-Based Optimization for Integer Programming

**DOI:** 10.1155/2014/672983

**Published:** 2014-06-03

**Authors:** Zhi-Cheng Wang, Xiao-Bei Wu

**Affiliations:** College of Electronics and Information Engineering, Tongji University, Shanghai 201804, China

## Abstract

Biogeography-based optimization (BBO) is a relatively new bioinspired heuristic for global optimization based on the mathematical models of biogeography. By investigating the applicability and performance of BBO for integer programming, we find that the original BBO algorithm does not perform well on a set of benchmark integer programming problems. Thus we modify the mutation operator and/or the neighborhood structure of the algorithm, resulting in three new BBO-based methods, named BlendBBO, BBO_DE, and LBBO_LDE, respectively. Computational experiments show that these methods are competitive approaches to solve integer programming problems, and the LBBO_LDE shows the best performance on the benchmark problems.

## 1. Introduction

An integer programming problem is a discrete optimization problem where the decision variables are restricted to integer values. In computer science and operations research, a remarkably wide range of problems, such as project scheduling, capital budgeting, goods distribution, and machine scheduling, can be expressed as integer programming problems [1–6]. Integer programming also has applications in bioinspired computational models such as artificial neural networks [7, 8].

The general form of an integer programming model can be stated as

(1) min⁡f(x→)s.t.x→∈S→⊆ZD,

where $\vec{x}$ is a *D*-dimensional integer vector, *Z* ^*D*^ is a *D*-dimensional discrete space of integers, and $\vec{S}$ is a feasible region that is not necessarily bounded. Any maximization version of integer programming problems can be easily transformed to a minimization problem.

One of the most well-known deterministic approaches for solving integer programming problems is the branch-and-bound algorithm [9]. It uses a “divide-and-conquer” strategy to split the feasible region into subregions, obtaining for each subregion a lower bound by ignoring the integrality constraints and checking whether the corresponding solution is a feasible one; if so, the current solution is optimum to the original problem; otherwise recursively split and tackle the subregions until all the variables are fixed to integers.

However, integer programming is known to be* NP*-hard [10], and thus the computational cost of deterministic algorithms increases very rapidly with problem size. In recent years, evolutionary algorithms (EA), which are stochastic search methods inspired by the principles of natural biological evolution, have attracted great attention and have been successfully applied to a wide range of computationally difficult problems. These heuristic algorithms do not guarantee finding the exact optimal solution in a single simulation run, but in most cases they are capable of finding acceptable solutions in a reasonable computational time.

Genetic algorithms (GA) are one of the most popular EA, but the encoding of the integer search space with fixed length binary strings as used in standard GA is not feasible for integer problems [11]. Many other heuristics, such as evolutionary strategy (ES) [12], particle swarm optimization (PSO) [13], and differential evolution (DE) [14], are initially proposed for continuous optimization problems. However, they can be adapted to integer programming by embedding the integer space into the real space and truncating or rounding real values to integers, and the applicability and performance of such approach are demonstrated by experimental studies.

Kelahan and Gaddy [15] conducted an early study that performs random search in integer spaces in the spirit of a (1 + 1)-ES; that is, at each iteration a child solution vector is generated by adding a random vector to the parent vector, and the better one between the parent and the child is kept for the next generation. Rudolph [11] developed a (*μ* + *λ*)-ES based algorithm, which uses the principle of maximum entropy to guide the construction of a mutation distribution for arbitrary search spaces.

Laskari et al. [16] studied the ability of PSO for solving integer programming problems. On their test problems, PSO outperforms the branch-and-bound method in terms of number of function evaluations (NFE), and PSO exhibits high success rates even in cases where the branch-and-bound algorithm fails. Improved versions of PSO, including the quantum-behaved PSO which is based on the principle of state superposition and uncertainty [17] and barebones PSO which is based on samples from a normal distribution and requires no parameter tuning [18], have also been applied and shown to be efficient alternatives to integer programming problems.

Omran and Engelbrecht [19] investigated the performance of DE in integer programming. They tested three versions of DE and found that the self-adaptive DE (SDE) requiring no parameter tuning is the most efficient and performs better than PSO.

In this paper, we propose three algorithms for integer programming based on a relatively new bioinspired method, namely, biogeography-based optimization (BBO). We modify the mutation operator of the original BBO to enhance its exploration or global search ability and adopt a local neighborhood structure to avoid premature convergence. Experimental results show that our methods are competitive approaches to solving integer programming problems.

## 2. Biogeography-Based Optimization

**Figure 1:**
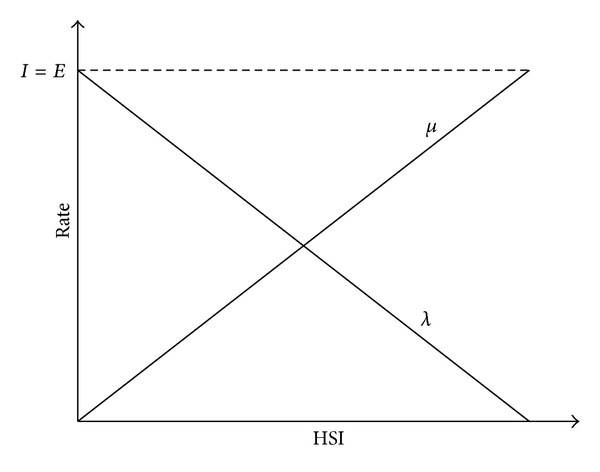
A linear model of emigration and immigration rates of a habitat.

Biogeography is the science of the geographical distribution of biological organisms over space and time. MacArthur and Wilson [20] established the mathematical models of island biogeography, which show that the species richness of an island can be predicted in terms of such factors as habitat area, immigration rate, and extinction rate. Inspired by this, Simon [21] developed the BBO algorithm, where a solution vector is analogous to a habitat, the solution components are analogous to a set of suitability index variables (SIVs), and the solution fitness is analogous to the species richness or habitat suitability index (HSI) of the habitat. Central to the algorithm is the equilibrium theory of island biogeography, which indicates that high HSI habitats have a high species emigration rate and low HSI habitats have a high species immigration rate. For example, in a linear model of species richness (as illustrated in Figure 1), a habitat *H*_*i*_'s immigration rate *λ*_*i*_ and emigration rate *μ*_*i*_ are calculated based on its fitness *f*_*i*_ as follows:

(2) $$\begin{array}{c} \lambda_{i}=I\left(\frac{f_{\max}-f_{i}}{f_{\max}-f_{\min}}\right)\\ \mu_{i}=E\left(\frac{f_{i}-f_{\min}}{f_{\max}-f_{\min}}\right), \end{array}$$

where *f*_max⁡_ and *f*_min⁡_ are, respectively, the maximum and minimum fitness values among the population and *I* and *E* are, respectively, the maximum possible immigration rate and emigration rate. However, there are other nonlinear mathematical models of biogeography that can be used for calculating the migration rates [22, 23].

Migration is used to modify habitats by mixing features within the population. BBO also has a mutation operator for changing SIV within a habitat itself and thus probably increasing diversity of the population. For each habitat *H*_*i*_, a species count probability *P*_*i*_ computed from *λ*_*i*_ and *μ*_*i*_ indicates the likelihood that the habitat was expected a priori to exist as a solution for the problem. In this context, very high HSI habitats and very low HSI habitats are both equally improbable, and medium HSI habitats are relatively probable. The mutation rate of habitat *H*_*i*_ is inversely proportional to its probability:

(3) $$\begin{array}{c} \pi_{i}=\pi_{\max}\left(1-\frac{P_{i}}{P_{\max}}\right), \end{array}$$

where *π*_max⁡_ is a control parameter and *P*_max⁡_ is the maximum habitat probability in the population.

Algorithm 1 describes the general framework of BBO for a *D*-dimensional global numerical optimization problem (where *l*_*d*_ and *u*_*d*_ are the lower and upper bounds of the *d*th dimension, respectively, and rand is a function that generates a random value uniformly distributed in [0,1]).

Typically, in Line 8 we can use a roulette wheel method for selection, the time complexity of which is *O*(*n*). It is not difficult to see that the complexity of each iteration of the algorithm is *O*(*n* ^2^ *D* + *nO*(*f*)), where *O*(*f*) is the time complexity for computing the fitness function *f*.

## 3. Biogeography-Based Heuristics for Integer Programming

In BBO, the migration operator provides good exploitation ability, while the broader exploration of the search space is mainly based on the mutation operator. Simon [21] suggested that *π*_max⁡_ should be set to a small value (about 0.01), which results in low mutation rates. However, when being applied to integer programming, we need to use higher mutation rates to improve the exploration of search space. According to our experimental studies, when *π*_max⁡_ is set to about 0.25~0.3, the BBO algorithm exhibits the best performance on integer programming problems.

Note that the migration operator does not violate the integer constraints, and the rounding of real values to integers is required only after mutations (Line 13 of Algorithm 1). Nevertheless, even using a higher mutation rate, the performance of BBO is far from satisfactory for integer programming. This is mainly because random mutation operator does not utilize any information of the population to guide the exploration of search space. In this work, we introduce two other mutation operators to BBO, which results in three variants of BBO for integer programming.

### 3.1. A Blended Mutation Operator

In the first variant, namely, BlendBBO, we use a blended mutation operator, which is motivated by the blended crossover operator used by Mühlenbein and Schlierkamp-Voosen [24] in GA and by Ma and Simon [25] in constrained optimization. In our approach, if a component of vector *H*_*i*_ is subject to mutate, we first select another vector *H*_*j*_ with probability ∝*μ*_*j*_ and then use the following equation to work out the new value of the component:

(4) $$\begin{array}{c} H_{i,d}=\text{round}\left(\alpha H_{i,d}+\left(1-\alpha \right)H_{j,d}\right), \end{array}$$

where *α* is a random value uniformly distributed in [0,1]. Note that if the *d*th dimension of the search space has a bound, (4) will never result in a value outside the bound.

Moreover, we employ an elitism mechanism in solution update (as used in ES [12, 26]): the migration operator always generates a new vector *H*_*i*_′ for each existing vector *H*_*i*_ (rather than directly changing *H*_*i*_); if *H*_*i*_′ is better than *H*_*i*_, no mutation will be applied and *H*_*i*_′ directly enters to the next generation; otherwise the mutation operator is applied to *H*_*i*_. This not only decreases the required NFE but also increases the convergence speed of the algorithm. The algorithm flow of BBO with the blended mutation is presented in Algorithm 2.

### 3.2. DE Mutation Operator

The second variant, namely, BBO_DE, replaces the random mutation operator with the mutation operator of DE, which mutates a vector component by adding the weighted difference between the corresponding components of two randomly selected vectors to a third one:

(5) $$\begin{array}{c} H_{i,d}=\text{round}\left(H_{r_{1},d}+F\left(H_{r_{2},d}-H_{r_{3},d}\right)\right), \end{array}$$

where *r*_1_, *r*_2_, and *r*_3_ are three unique randomly selected habitat indices that are different to *i*, and *F* is a constant scaling coefficient.

DE is well known for its good exploration ability, and the combination of BBO migration and DE mutation achieves a good balance between exploitation and exploration. BBO_DE also uses our new solution update mechanism described above. Therefore, the algorithm flow of BBO_DE simply replaces Lines 15 and 16 of Algorithm 2 with the DE mutation operation described by (5).

### 3.3. Local Topology of the Population

The original BBO uses a fully connected topology; that is, all the individual solutions are directly connected in the population and can migrate with each other. But such a global topology is computationally intensive and is prone to premature convergence. To overcome this problem, our third variant replaces the global topology with a local one. One of the simplest local topologies is the ring topology, where each individual is directly connected to two other individuals [27, 28]. But here we employ a more generalized local topology, the random topology, where each individual has *K* immediate neighbors that are randomly selected from the population and *K* is a control parameter [28].

In consequence, whenever an individual vector *H*_*i*_ is to be immigrated, the emigrating vector is chosen from its neighbors rather than the whole population, based on the migration rates. The neighborhood structure can be saved in an *n* × *n* matrix *L*: if two habitats *H*_*i*_ and *H*_*j*_ are directly connected then *L*(*i*, *j*) = 1; otherwise *L*(*i*, *j*) = 0. It is easy to see that the complexity of each iteration of the algorithm is *O*(*nKD* + *nO*(*f*)).

Storn and Price [14] have proposed several different strategies on DE mutation. The scheme of (5) is denoted as DE/rand/1. Another scheme is named DE/best/1, which always chooses the best individual of the population as *H*_*r*_1__ in (5). Omran et al. [29] extended it to the DE/lbest/1 scheme, which uses a ring topology and always chooses the better neighbor of the vector to be mutated.

In our approach, BBO migration and DE mutation share the same local random topology. That is, each *H*_*i*_ individual has *K* neighbors, and at each time an *H*_*j*_ is chosen from the neighbors with probability ∝*μ*_*j*_ to participate in the mutation such that

(6) $$\begin{array}{c} H_{i,d}=\text{round}\left(H_{j,d}+F\left(H_{r_{2},d}-H_{r_{3},d}\right)\right). \end{array}$$

Moreover, if the current best solution has not been improved after every *n*_*p*_ generation (where *n*_*p*_ is a predefined constant), we reset the neighborhood structure randomly.

The third variant is named LBBO_LDE, and it also uses the same solution update mechanism as the previous two variants.

## 4. Computational Experiments

We test the three variants of BBO on a set of integer programming benchmark problems, which are taken from [16, 30, 31] and frequently encountered in the relevant literature. The details of the benchmark problems are described in the Appendix. For comparison, we also implement the basic BBO, DE, and SDE [19] for integer programming. The branch-and-bound method is not included for comparison, because it has shown that DE outperforms branch-and-bound on most test problems [16, 19].

For all the six algorithms, we use the same population size *n* = 50 and run them on each problem for 40 times with different random seeds. The migration control parameters are set as *I* = *E* = 1 for BBO, BlendBBO, BBO_DE, and LBBO_LDE, and the mutation control parameter *π*_max⁡_ is set to 0.01 for BBO and 0.25 for BlendBBO (BBO_DE and LBBO_LDE do not use this parameter). Empirically, the neighborhood size *K* and the threshold of nonimprovement generations *n*_*p*_ are both set to 3 for LBBO_LDE. The other parameters with regard to DE and SDE are set as suggested in [14, 32].

**Figure 2:**
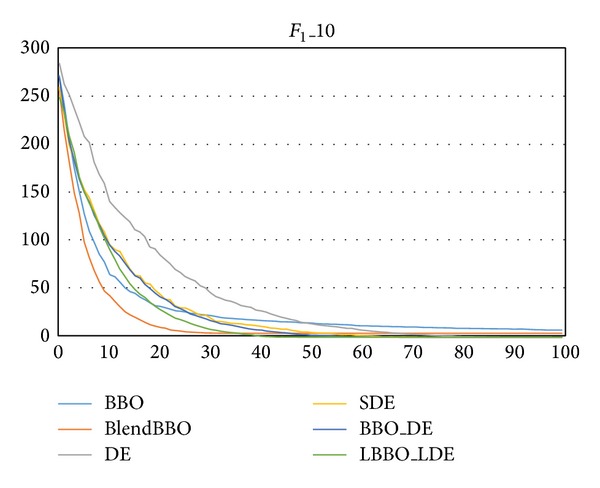
The convergence curves of the algorithms on *F*_1_ in 10 dimensions, where the vertical axis represents the objective value and the horizontal axis represents the number of generations.

The first two problems *F*_1_ and *F*_2_ are high-dimensional problems. For *F*_1_, we, respectively, consider it in 10 and 30 dimensions.  Table 1 presents the success rates (SR) and required NFE of the algorithms to achieve the optimum in 10 dimensions, and Figure 2 presents the corresponding convergence curves of the algorithms. As we can see, the original BBO fails to solve the problem, and the SR of BlendBBO is only 20%. The four algorithms utilizing the DE mutation operator can guarantee the optimal result on the 10-dimensional problem, among which LBBO_LDE shows the best performance, and the other three algorithms have similar performance, but the result of BBO_DE is slightly better than DE and SDE.

**Figure 3:**
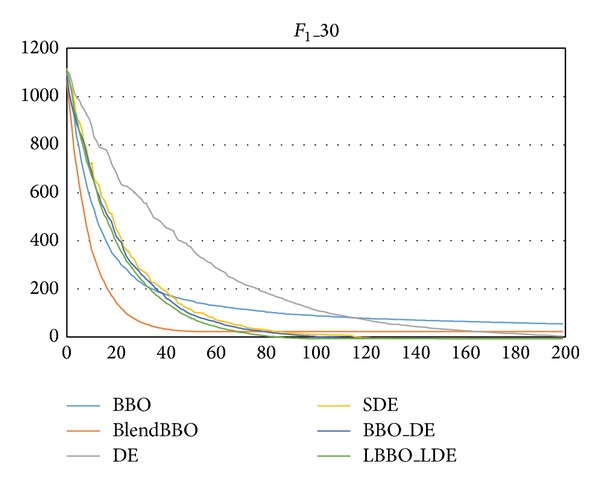
The convergence curves of the algorithms on *F*_1_ in 30 dimensions, where the vertical axis represents the objective value and the horizontal axis represents the number of generations.

Table 2 and Figure 3, respectively, present the results and the convergence curves of the algorithms on *F*_1_ in 30 dimensions. On this high-dimensional problem, BBO, BlendBBO, and DE all fail to obtain the optimum, SDE and BBO_DE, respectively, have SR of 85% and 90% for obtaining the optimum, and only our LBBO_LDE can always guarantee the optimum.

From the convergence curves we can also find that the BBO algorithm converges very fast at the early stage, but thereafter its performance deteriorates because it is ineffective to explore other potentially promising areas of the search space. By combining with the DE mutation operator, our hybrid BBO methods inherit the fast convergence speed of BBO, at the same time taking advantage of the exploration ability of DE.

**Figure 4:**
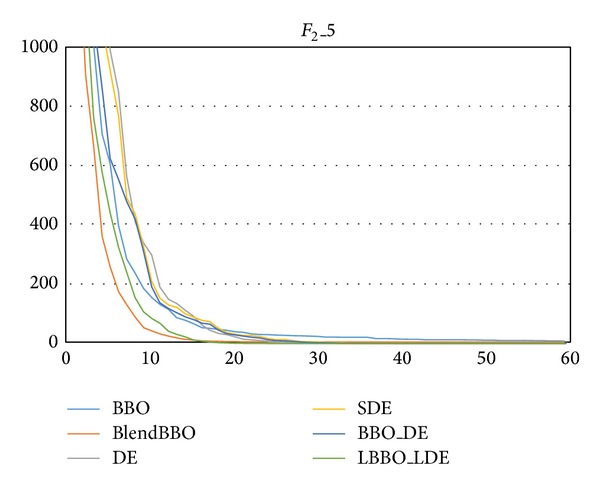
The convergence curves of the algorithms on *F*_2_ in 5 dimensions, where the vertical axis represents the objective value and the horizontal axis represents the number of generations.

**Figure 5:**
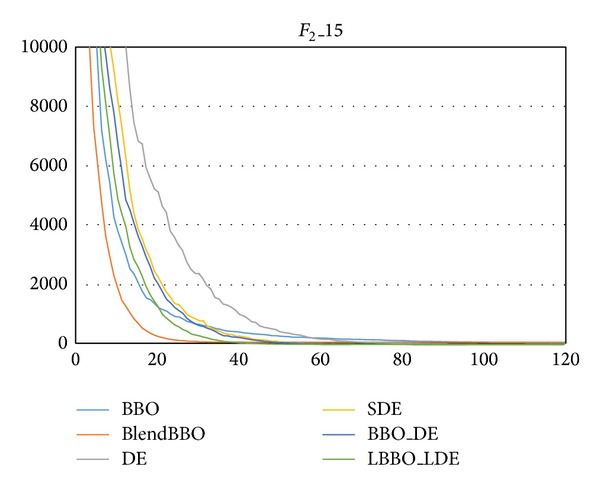
The convergence curves of the algorithms on *F*_2_ in 15 dimensions, where the vertical axis represents the objective value and the horizontal axis represents the number of generations.

For *F*_2_, we, respectively, consider it in 5 and 15 dimensions, the experimental results of which are, respectively, presented in Tables 3 and 4 and the convergence curves of which are presented in Figures 4 and 5. The results are similar to those of *F*_1_: for the low dimensional problem, SDE, BBO_DE, and LBBO_LDE are efficient; for the high-dimensional problem, only LBBO_LDE can guarantee the optimum; the performance LBBO_LDE is the best while that of BBO is the worst; SDE performs better than DE and BBO_DE performs slightly better than SDE, and BlendBBO outperforms BBO but is worse than the algorithms with the DE mutation operator.

**Figure 6:**
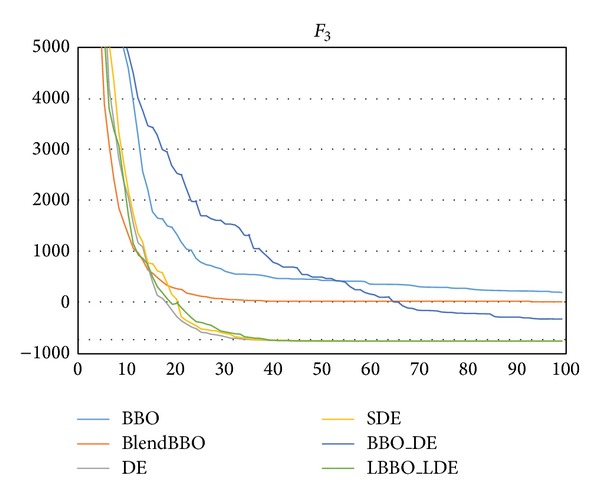
The convergence curves of the algorithms on *F*_3_, where the vertical axis represents the objective value and the horizontal axis represents the number of generations.

*F*_3_ is a 5-dimensional problem more difficult than *F*_2_. As we can see from the results shown in Table 5, BBO and BlendBBO always fail on the problem, and DE, SDE, and LBBO_LDE can guarantee the optimum. The required NFE of DE is slightly better than SDE and LBBO_LDE, but LBBO_LDE converges faster than DE, as shown in Figure 6.

**Figure 7:**
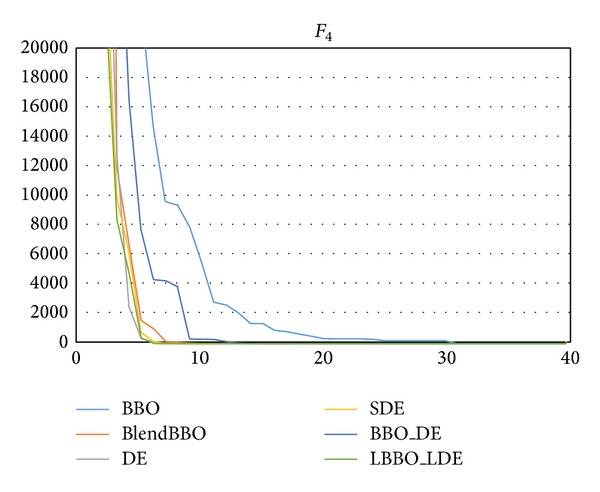
The convergence curves of the algorithms on *F*_4_, where the vertical axis represents the objective value and the horizontal axis represents the number of generations.

*F*_4_ is a relatively easy problem, on which even the worst BBO has an SR of 75%, and all the other algorithms can guarantee the optimum. LBBO_LDE is the best one in terms of both NFE and convergence speed, as shown in Table 6 and Figure 7.

**Figure 8:**
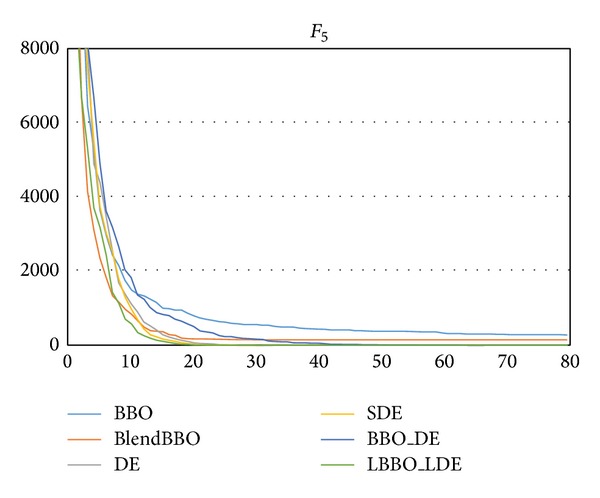
The convergence curves of the algorithms on *F*_5_, where the vertical axis represents the objective value and the horizontal axis represents the number of generations.

**Figure 9:**
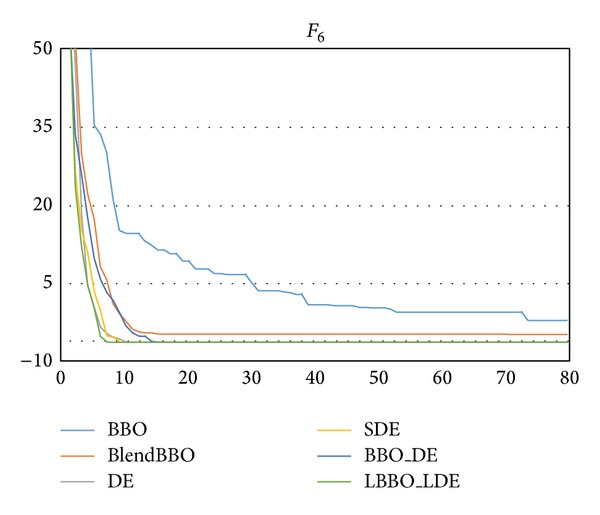
The convergence curves of the algorithms on *F*_6_, where the vertical axis represents the objective value and the horizontal axis represents the number of generations.

**Figure 10:**
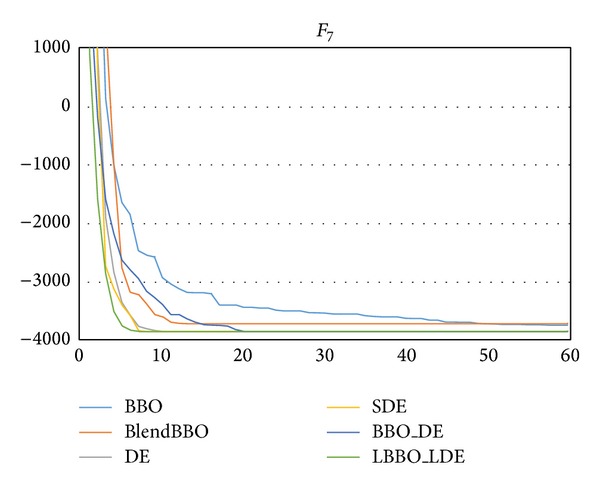
The convergence curves of the algorithms on *F*_7_, where the vertical axis represents the objective value and the horizontal axis represents the number of generations.

The remaining three test problems are also relatively easy. The experimental results are presented in Tables 7, 8, and 9, and the convergence curves are shown in Figures 8, 9, and 10, respectively. As we can clearly see, the four algorithms with the DE mutation operator can always obtain the optima on these problems, and LBBO_LDE shows the best performance.

In summary, our LBBO_LDE outperforms the other algorithms on all of the test problems. Generally speaking, the original BBO converges fast at first, but it is easy to be trapped by the local optima. BlendBBO alleviates the dilemma to a certain degree, but the DE mutation operator is more effective than the blended mutation operator, as demonstrated by our experimental results. By combining BBO and DE, the BBO_DE algorithm provides an efficient alternative to popular methods such as SDE. The local topology used in LBBO_LDE further improves the search ability and suppresses the premature convergence, especially on high-dimensional problems where the performance of DE and SDE deteriorates quickly. Therefore, LBBO_LDE is a very competitive heuristic method for solving integer programming problem.

## 5. Conclusion

In this paper we develop three algorithms for integer programming based on the BBO heuristic. The BlendBBO uses a blended mutation operator, BBO_DE integrates the DE mutation operator, and LBBO_LDE further uses a local neighborhood structure for selecting individuals for migration and mutation. Experimental results show that LBBO_LDE has the best performance on a set of benchmark integer programming problem.

In general, the LBBO_LDE algorithm with local neighborhood size *K* of 3~5 is efficient on the test problem, but none of the values can provide the best performance on all the problems. Currently we are studying a mechanism that dynamically adjusts the neighborhood size as well as other control parameters according to the search state [33]. Moreover, the test problems considered in the paper only have bounds for decision variables but do not include other constraints, and we are extending the proposed approach to solve more complex constrained optimization problems, including multiobjective ones [34–36]. We also believe that our approach can be adapted to effectively handle other kinds of combinatorial optimization problems, such as 0-1 integer programming and permutation-based optimization.

## Figures and Tables

**Algorithm 1:**
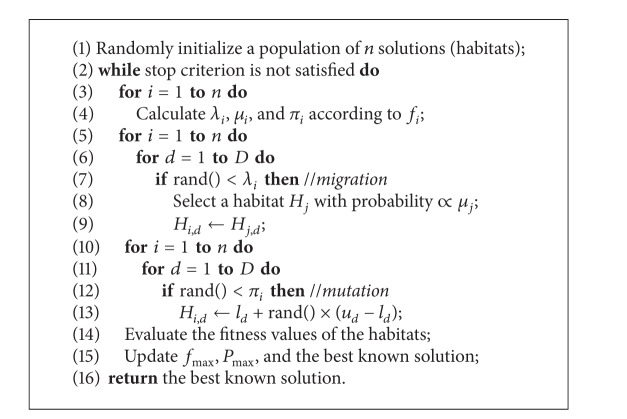
The original BBO algorithm.

**Algorithm 2:**
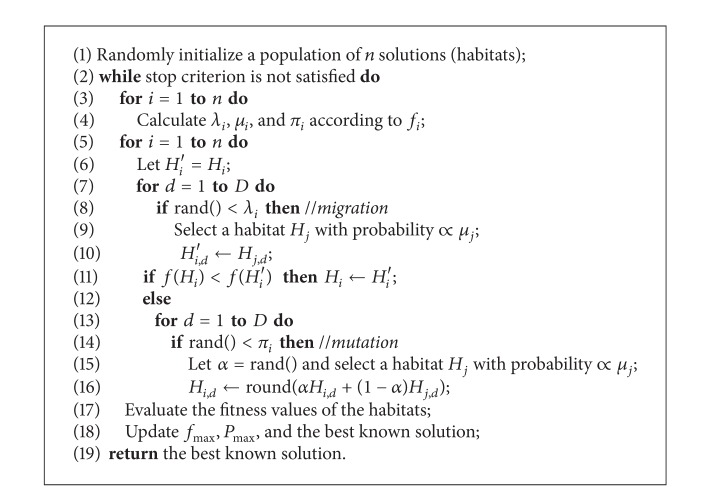
The BBO with the blended mutation for integer programming.

**Table 1:** SR and required NFE of the algorithms on *F*_1_ in 10 dimensions.

Method	SR	Best	Worst	Mean	Std
BBO	0%	NA	NA	NA	NA
BlendBBO	20%	1946	2215	2080.50	190.21
DE	100%	3200	4100	3777.50	239.78
SDE	100%	3050	4050	3762.40	265.23
BBO_DE	100%	3102	3964	3674.80	269.07
LBBO_LDE	100%	2061	2694	2493.75	145.59

**Table 2:** SR and required NFE of the algorithms on *F*_1_ in 30 dimensions.

Method	SR	Best	Worst	Mean	Std
BBO	0%	NA	NA	NA	NA
BlendBBO	0%	NA	NA	NA	NA
DE	0%	NA	NA	NA	NA
SDE	85%	7850	11350	9301.00	1130.10
BBO_DE	90%	6765	9752	8204.25	920.39
LBBO_LDE	100%	5923	7071	6471.60	272.29

**Table 3:** SR and required NFE of the algorithms on *F*_2_ in 5 dimensions.

Method	SR	Best	Worst	Mean	Std
BBO	10%	3663	5168	4415.50	1064.20
BlendBBO	55%	1154	1545	1363.36	121.94
DE	95%	1500	2100	1797.37	188.91
SDE	100%	1450	2600	2022.35	368.17
BBO_DE	100%	1773	2669	2336.65	258.22
LBBO_LDE	100%	1058	1898	1451.20	214.48

**Table 4:** SR and required NFE of the algorithms on *F*_2_ in 15 dimensions.

Method	SR	Best	Worst	Mean	Std
BBO	0%	NA	NA	NA	NA
BlendBBO	2.5%	4815	4815	4815	NA
DE	95%	5300	6650	5978.95	335.95
SDE	95%	5000	6400	5698.32	389.24
BBO_DE	97.5%	5030	6210	5639.11	362.69
LBBO_LDE	100%	3488	4528	4188.30	300.77

**Table 5:** SR and required NFE of the algorithms on *F*_3_.

Method	SR	Best	Worst	Mean	Std
BBO	0%	NA	NA	NA	NA
BlendBBO	0%	NA	NA	NA	NA
DE	100%	2050	2950	2490.00	262.38
SDE	100%	2500	4050	3260.00	638.05
BBO_DE	10%	4810	5246	5028.00	308.30
LBBO_LDE	100%	1758	4181	2958.85	849.24

**Table 6:** SR and required NFE of the algorithms on *F*_4_.

Method	SR	Best	Worst	Mean	Std
BBO	75%	477	3429	1726.87	1020.19
BlendBBO	100%	258	552	424.50	85.34
DE	100%	300	650	420.00	93.75
SDE	100%	250	600	520.00	129.65
BBO_DE	10%	59	1058	632.30	277.77
LBBO_LDE	100%	236	525	400.60	72.20

**Table 7:** SR and required NFE of the algorithms on *F*_5_.

Method	SR	Best	Worst	Mean	Std
BBO	0%	NA	NA	NA	NA
BlendBBO	25%	1223	2676	1842	692.53
DE	100%	1200	1700	1445.00	140.39
SDE	100%	1100	1750	1550.50	196.45
BBO_DE	100%	2473	4498	3681.25	620.36
LBBO_LDE	100%	1012	1874	1532.35	249.46

**Table 8:** SR and required NFE of the algorithms on *F*_6_.

Method	SR	Best	Worst	Mean	Std
BBO	45%	140	1989	1150.22	678.16
BlendBBO	80%	178	635	455.81	128.81
DE	100%	200	550	392.50	140.39
SDE	100%	200	500	405.20	103.72
BBO_DE	100%	196	995	708.50	198.38
LBBO_LDE	100%	183	511	410.05	86.37

**Table 9:** SR and required NFE of the algorithms on *F*_7_.

Method	SR	Best	Worst	Mean	Std
BBO	60%	167	3403	1714.75	986.18
BlendBBO	70%	262	721	459.50	149.00
DE	100%	350	600	480.00	76.78
SDE	100%	300	650	451.00	89.33
BBO_DE	100%	479	1327	978.40	311.11
LBBO_LDE	100%	249	614	389.45	96.08

## Appendix

### Integer Programming Benchmark Problems

Consider

(A.1) F1(x→)=∑i=1Dxi, x→∗=(0)D, F1(x→∗)=0,F2(x→)=∑i=1Dxi2, x→∗=(0)D, F2(x→∗)=0,F3(x→)=−(15,27,36,18,12)x→⊤+x→(35−20−1032−10−2040−6−3132−10−611−6−1032−31−638−20−1032−10−2032)x→⊤,x→∗=(0,11,22,16,6) or  x→∗=(0,12,23,17,6),F3(x→∗)=−737,F4(x→)=(9x12+2x22−11)2+(3x1+4x22−7)2,x→∗=(1,1), F4(x→∗)=0,F5(x→)=(x1+10x2)2+5(x3−x4)2+(x2−2x3)4+10(x1−x4)4,x→∗=(0)4, F5(x→∗)=0,F6(x→)=2x12+3x22+4x1x2−6x1−3x2,  x→∗=(2,−1), F6(x→∗)=−6,F7(x→)=−3803.84−138.08x1−232.93x2+123.08x12+203.64x22+182.25x1x2,x→∗=(0,1), F7(x→∗)=3833.12.

In the above problems, the ranges of variables are all set as $\vec{x}\in {\left[-100,100\right]}^{D}$.

## References

[1] Sofianopoulou S (1992). The process allocation problem: a survey of theapplication of graph-theoretic and integer programming approaches. *Journal of the Operational Research Society*.

[2] Whitacre JM (2011). Recent trends indicate rapid growth of nature-inspired optimization in academia and industry. *Computing*.

[3] Zheng Y, Xue J (2010). A problem reduction based approach to discrete optimization algorithm design. *Computing*.

[4] Zheng YJ, Chen SY, Lin Y, Wang WL (2013). Bio-inspired optimizationof sustainable energy systems: a review. *Mathematical Problemsin Engineering*.

[5] Zheng YJ, Ling HF, Shi HH, Chen HS, Chen SY (2014). Emergencyrailway wagon scheduling by hybrid biogeography-based optimization. *Computers & Operations Research*.

[6] Zheng YJ, Ling HF, Xue JY (2014). Ecogeography-based optimization: enhancing biogeography-based optimization with ecogeographic barriers and differentiations. *Computers & Operations Research*.

[7] Dua V (2010). A mixed-integer programming approach for optimal configuration of artificial neural networks. *Chemical Engineering Research and Design*.

[8] Tan Y, Wang J, Zurada JM (2001). Nonlinear blind source separation using a radial basis function network. *IEEE Transactions on Neural Networks*.

[9] Nemhauser G, Wolsey L (1988). *Integer and Combinatorial Optimization*.

[10] Garey MR, Johnson DS, Sethi R (1976). The complexity of flowshop and jobshop scheduling. *Mathematics of Operations Research*.

[11] Rudolph G, Davidor Y, Schwefel HP, Männer R (1994). An evolutionary algorithm for integer programming. *Parallel Problem Solving from Nature*.

[12] Beyer HG, Schwefel HP (2002). Evolution strategies—a comprehensive introduction. *Natural Computing*.

[13] Kennedy J, Eberhart R Particle swarm optimization.

[14] Storn R, Price K (1997). Differential evolution—a simple and efficient heuristic for global optimization over continuous spaces. *Journal of Global Optimization*.

[15] Kelahan R, Gaddy J (1978). Application of the adaptive random searchto discrete and mixed integer optimization. *International Journal for Numerical Methods in Engineering*.

[16] Laskari EC, Parsopoulos KE, Vrahatis MN Particle swarm optimizationfor integer programming.

[17] Liu J, Sun J, Xu W, King I, Wang J, Chan LW, Wang D (2006). Quantum-behaved particle swarm optimizationfor integer programming. *Neural Information Processing*.

[18] Omran MGH, Engelbrecht A, Salman A Barebones particle swarm for integer programming problems.

[19] Omran MGH, Engelbrecht AP Differential evolution for integer programming problems.

[20] MacArthur R, Wilson E (1967). *The Theory of Biogeography*.

[21] Simon D (2008). Biogeography-based optimization. *IEEE Transactions on Evolutionary Computation*.

[22] Ma H (2010). An analysis of the equilibrium of migration models for biogeography-based optimization. *Information Sciences*.

[23] Ma H, Simon D (2011). Analysis of migration models of biogeography-based optimization using Markov theory. *Engineering Applications of Artificial Intelligence*.

[24] Mühlenbein H, Schlierkamp-Voosen D (1993). Predictive models for thebreeder genetic algorithm I. Continuous parameter optimization. *Evolutionary Computation*.

[25] Ma H, Simon D (2011). Blended biogeography-based optimization for constrained optimization. *Engineering Applications of Artificial Intelligence*.

[26] Du D, Simon D, Ergezer M Biogeography-based optimization combined with evolutionary strategy and immigration refusal.

[27] Li X (2010). Niching without niching parameters: particle swarm optimization using a ring topology. *IEEE Transactions on Evolutionary Computation*.

[28] Zheng YJ, Ling HF, Wu XB, Xue JY (2014). Localized biogeographybasedoptimization. *Soft Computing*.

[29] Omran MG, Engelbrecht AP, Salman A Using neighborhood topologies with differential evolution.

[30] Glankwahmdee A, Liebman JS, Hogg GL (1979). Unconstrained discrete nonlinear programming. *Engineering Optimization*.

[31] Rao S (1996). *Engineering Optimization—Theory and Practice*.

[32] Omran M, Salman A, Engelbrecht A, Hao Y, Liu J, Wang Y (2005). Self-adaptive differential evolution. *Computational Intelligence and Security*.

[33] Zheng YJ, Ling HF, Guan Q (2012). Adaptive parameters for a modifiedcomprehensive learning particle swarm optimizer. *Mathematical Problemsin Engineering*.

[34] Zheng YJ, Song Q, Chen SY (2013). Multiobjective fireworks optimizationfor variable-rate fertilization in oil crop production. *Applied Soft Computing*.

[35] Zheng YJ, Chen SY (2013). Cooperative particle swarm optimization formultiobjective transportation planning. *Applied Intelligence*.

[36] Zheng Y, Ling H, Xue J, Chen S (2014). Population classificationin fire evacuation: a multiobjective particle swarm optimization approach. *IEEE Transactions on Evolutionary Computation*.

